# The melt content of the low velocity layer atop the mantle transition zone: Theory and method of calculation

**DOI:** 10.1016/j.mex.2019.11.024

**Published:** 2019-11-27

**Authors:** Maining Ma, Jikai Zhang, Xiaoya Zhou, Zhishuang Xu

**Affiliations:** a Key Laboratory of Computational Geodynamics, College of Earth and Planetary Sciences, University of Chinese Academy of Sciences, Beijing, China; b China Earthquake Networks Center, Beijing, China

**Keywords:** The computational methods for melt volume fractions, Melt content, Low velocity layer, The equilibrium geometry model, Parameter selection

## Abstract

The melt content is significant characteristic for the low velocity layer, so it is very necessary to set up the quantitative relationship between the low velocity anomaly and the melt fraction. We describe the computational methods for melt volume fractions and discussed the parameter selections for the theoretical computations.

• We discuss the seismic wave velocity characteristics and the equilibrium geometry model in the partial melting system.
• Equations for computing the elastic properties atop the LVL are presented.
• Parameter selection of the equilibrium geometry model is shown.

## Graphical abstract

**Figure:**
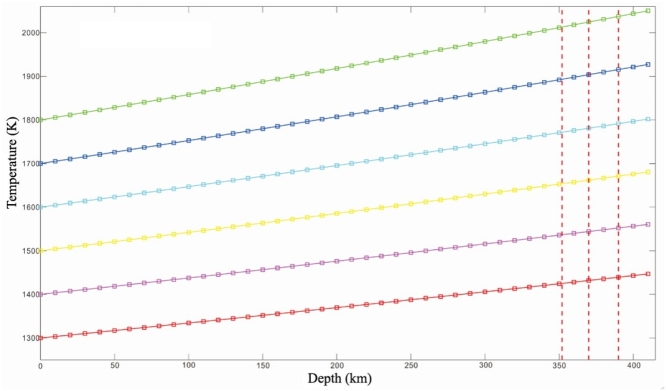

**Specification Table**

**Table:** 

Subject Area:	Earth and Planetary Sciences
More specific subject area:	*Physics of Earth’s Interior*
Method name:	The computational methods for melt volume fractions
Name and reference of original method:	1 Hier-Majumder, S., Abbott, M. E., 2010. Influence of dihedral angle on the seismic velocities in partially molten rocks. Earth Planet. Sci. Lett. 299, 23–32. 2 Hier-Majumder, S., Courtier, A., 2011. Seismic signature of small melt fraction atop the transition zone. Earth Planet. Sci. Lett. 308, 334–342. 3 Hier-Majumder, S., Keel, E. B., Courtier, A. M., 2014. The influence of temperature, bulk composition, and melting on the seismic signature of the low-velocity layer above the transition zone. J. Geophys. Res. Solid Earth 119, 971–983. 4 Hier-Majumder, S., Ricard, Y., Bercovici, D., 2006. Role of grain boundaries in magma migration and storage. Earth Planet. Sci. Lett. 248, 735–749.
Resource availability:	N.A.

## Method details

### Seismic wave velocity characteristics in the partial melting systems

The LVL is one partial melting system there exists melt and solid simultaneously. The quantitative effect of melt on seismic wave velocity is of fundamental importance to consider the seismic detectability of melt. The melt has two effects on seismic wave velocity: the first one is a direct effect due to the contrast in elastic properties between melt and solid, called the poroelastic effect (e.g., McCarthy and Takei, [1]), and the second one is indirect effect and attributed to enhanced attenuation and dispersion by melt, called the anelastic effect (e.g., Karato and Spetzler, [2]).

(1) The poroelastic effect

The poroelastic effect is related to the fractions of melt and solid [3,4]. Based on the two-phase continuum mechanics or poroelastic theory (e.g., Biot, [5]; Johnson and Plona, [6]), the effect of melt on the ratio between S- and P- wave velocities mainly depends on the bulk modulus (*K_b_*) and shear modulus (*N*) of the solid skeleton. So in this approach, the heterogeneity of the liquid pressure in the pore size scales is not taken into account [4].

The velocity variations of the shear and primary waves caused by a liquid phase is given by Takei, [4]

(1) $$\frac{V_{S}}{V_{S}^{0}}=\frac{\sqrt{N/\mu }}{\sqrt{\bar{\rho }/\rho }},$$

(2) $$\frac{V_{P}}{V_{P}^{0}}=\frac{\sqrt{K_{eff}/k+\left(4\gamma /3\right)N/\mu }}{\sqrt{1+4\gamma /3}\sqrt{\bar{\rho }/\rho }},$$

where

(3) $$\frac{K_{eff}}{k}=\frac{K_{b}}{k}+\frac{{\left(1-K_{b}/k\right)}^{2}}{1-\phi -K_{b}/k+\phi k/k_{f}},$$

(4) $$\gamma =\mu /k=\left\{3\left(1-2\upsilon \right)\right\}/\left\{2\left(1+\upsilon \right)\right\},$$

(5) $$\bar{\rho }=\left(1-\phi \right)\rho +\phi \rho_{f},$$

where $V_{S}^{0}$, $V_{P}^{0}$, *k*, *μ*, *υ*, *ρ* represent the shear and primary wave velocities, bulk and shear modulus, Poisson’s ratio, and density, respectively, of the solid phase. *k_f_* and *ρ_f_* are the bulk modulus and density of the liquid phase. *K_eff_* is the effective bulk modulus for the solid-liquid aggregate.

(2) The anelastic effect

The anelastic effect is related to the attenuation and dispersion (e.g., Karato and Spetzler, [2]). The liquid flow in rock pores effected by seismic waves is the most important reason leading to the anelastic effect. In the seismic wave frequency domain, the melt-bearing system corresponds to the wider attenuation peak than the solid system [7,8]. Background attenuation shows the broad absorption band behavior which dues to the diffusionally-accommodated grain boundary sliding [[9], [10], [11], [12], [13]]. Jackson et al. [7] showed that the causative mechanism was the elastically-accommodated grain boundary sliding. However, this attenuation peak has not been found in other similar experiments.

In summary, there is no widely approved physical model about the anelastic effect on seismic waves in upper mantle conditions (high temperature and low stress). So in this paper, only the poroelastic effect is taken into account.

### The equilibrium geometry model in the partial melting system

**Fig. 1:**
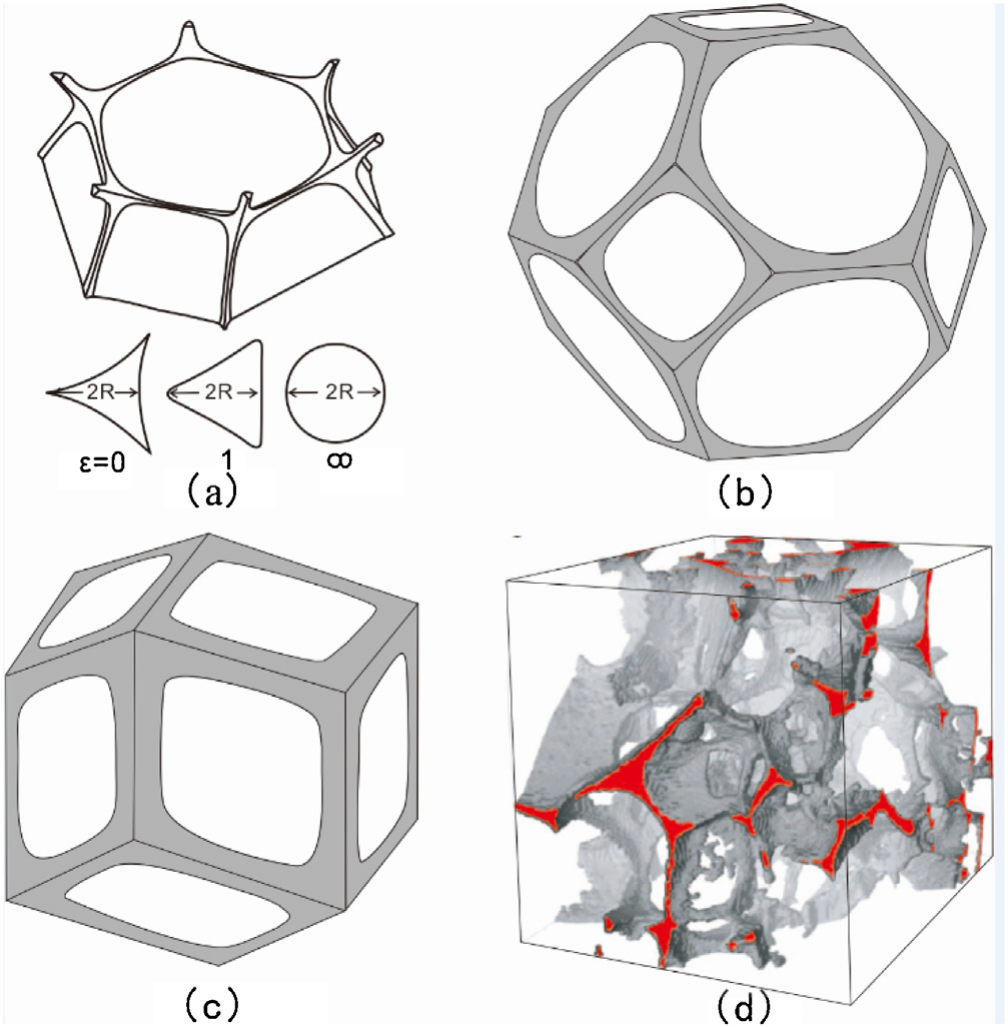
Common pore geometry models (a, b, c) and three-dimensional melt distribution (d) [16,17].

Selection of the pore geometry is vitally important to understand the characteristics of seismic wave velocities It is necessary to make specific assumptions and simplify the pore geometry carefully in practical calculations of *K_b_* and *N*. Comparing with many other simplified models, including the crack model [14], the oblate spheroid model [15], and the tube model [16] (Fig. 1a), the equilibrium geometry model proposed by Takei [4] is the best approximation to the real morphology of melt-bearing aggregate [17] (Fig. 1d).

The equilibrium geometry model is an equilibrium structure of the system with minimum surface free energy, and the free energy is closely related to melt distribution. In the partial melting system described by the model, the framework is formed by a certain coordination number 12 or 14 between solid particles, while melt distributes in inter-granular pores and the skeleton is formed by the solid framework with vacuum pores (e.g., Fig. 1b,c). If the effect of crystal anisotropy is negligible, the equilibrium pore geometry can be almost uniquely determined by the porosity and the dihedral angle [18], while the dihedral angle is determined by the ratio of solid-solid and solid-liquid interfacial energies [e.g., von Bargen and Waff, [19]].

For the equilibrium geometry model, the contiguity *ϕ* is the essential geometrical factor which can determine the macroscopic elasticity of the skeletal framework, such as the bulk and shear moduli of the skeleton, *K_b_* and *N*. The contiguity is the ratio of the area of each grain in contact with the neighboring grains to the total surface area [4]. In partially molten rocks, the melt fraction, termed the “disaggregation melt fraction” or the “rheologically critical melt fraction” is marked by a sharp reduction in viscosity [20]. When the melt fraction (*ϕ*) is between 0 and the disaggregation melt fraction, the contiguity depends on the melt fraction and the dihedral angle. When the melt fraction is higher than the disaggregation melt fraction, the system structure changes from particle to liquid support, and particles suspend isolated in a liquid [19,21]. The typical disaggregation melt fraction is 20∼30 vol. %, which is usually much higher than the situation in the mantle [20,22].

Quantitative relationship of the contiguity and melt fraction and the dihedral angle depends on the geometry of grains packing, such as the tetrakaidekahedral model [19] and the rhombic dodecahedral model [4]. Takei [4] combined the two models, and the theoretical values were in good agreement with the experimental results. So as the same, in this paper, we first derived the relationship between *ϕ* and a certain *φ* and *θ* from the tetrakaidekahedral model, then get the elastic modulus of the skeletal framework from the rhombic dodecahedral model.

(1) *ϕ* (*φ*, *θ*) [19]

(6) $$\varphi \left(\phi ,\theta \right)=2A_{\text{SS}}/\left(A_{\text{SL}}+2A_{\text{SS}}\right),$$

where *A*_SS_ and *A*_SL_ are solid-solid and solid-liquid interfacial area, respectively. Because solid-solid interfaces is shared by two solids, it's twice as much here.

(7) $$A_{\text{SS}}={\bar{A}}_{\text{SS}}-A_{\text{SS}}^{\text{V}}\cdot d,$$

where ${\bar{A}}_{SS}\approx \pi$ is the difference of grain boundary areas between dry system and melt-containing system [23].

(8) $$A_{\text{SL}}=A_{\text{SL}}^{\text{V}}\cdot d,$$

where $A^{V}\cdot d$ is the dimensionless surface areas per unit volume, also be expressed as:

(9) $$A^{V}\cdot d=b\phi^{p},$$

where *b* and *p* depend on *θ* (in degrees) as follows (when θ<60°):

(10) $$b=b_{2}\theta^{2}+b_{1}\theta +b_{0},$$

(11) $$p=p_{2}\theta^{2}+p_{1}\theta +p_{0}.$$

The coefficients *b_i_* and *p_i_* are listed in Table 1.

(2) *K_b_* and *N* [4]

**Table 1:** Coefficients for the interfacial area of the tetrakaidekahedral model (von Bargen and Waff, 1986).

*i*	*b_i_*	*p_i_*
	solid-solid interfaces	
0	8.1600	0.42397
1	−7.7102*10^-2^	9.9541*10^−4^
2	1.0353*10^−3^	8.6645*10^−6^
	solid-liquid interfaces	
0	12.8628	0.42786
1	−7.8562*10^-2^	8.6319*10^−5^
2	1.0043*10^−3^	2.4052*10^−5^

The bulk and shear moduli of the skeleton can be expressed as:

(12) $$K_{b}=\left(1-\phi \right)k_{sk}$$

(13) $$N=\left(1-\phi \right)\mu_{sk}$$

where $k_{sk}$ 、 $\mu_{sk}$ are the bulk and shear moduli of liquid-free system with solid skeleton only, and they are on the function of *ϕ*, and can be determined from the rhombic dodecahedral model.

(14) $$\frac{k_{sk}}{k}=g\left(\varphi \right)=1-{\left(1-\varphi \right)}^{n_{k}}$$

(15) $$\frac{\mu_{sk}}{\mu }=h\left(\varphi \right)=1-{\left(1-\varphi \right)}^{n_{\mu }}$$

where *n_k_* and *n_μ_* are also parameters associated with *ϕ*, and can be expressed as:

(16) $$n_{k}=a_{1}\varphi +a_{2}\left(1-\varphi \right)+a_{3}\varphi {\left(1-\varphi \right)}^{3/2}$$

(17) $$n_{\mu }=b_{1}\varphi +b_{2}\left(1-\varphi \right)+b_{3}\varphi {\left(1-\varphi \right)}^{2}$$

where the coefficients *a_i_* and *b_i_* (*i* = 1, 2, 3) are given by polynomial functions of the intrinsic Poisson’s ratio of the solid phase, *υ*, as

(18) $$a_{i}=\sum_{j=0}^{3}{\hat{a}}_{ij}\upsilon^{j}$$

(19) $$b_{i}=\sum_{j=0}^{2}{\hat{b}}_{ij}\upsilon^{j}$$

where ${\hat{a}}_{ij}\left(i=1-3,j=0-3\right),{\hat{b}}_{ij}\left(i=1-3,j=0-2\right)$ are given in Table 2 [4]. Eqs. (14), (15), (16), (17), (18), (19) were obtained by fitting the numerical results calculated for 0.1≤*ϕ*≤1 and 0.05≤*υ*≤0.45. When *K_b_* and *N* of the equilibrium geometry model are substituted into the equation of the poroelastic effect, the low velocity anomalies caused by melts will be obtained.

**Table 2:** Fitting parameters for *k*_sk_ and *μ*_sk_ of the rhombic dodecahedral model [4].

	*k_sk_*			*μ_sk_*		
*j*	${\hat{a}}_{1j}$	${\hat{a}}_{2j}$	${\hat{a}}_{1j}$	${\hat{b}}_{1j}$	${\hat{b}}_{2j}$	${\hat{b}}_{3j}$
0	1.8625	4.5001	−5.6512	1.6122	4.5869	−7.5395
1	0.52594	−6.1551	6.9159	0.13527	3.6086	−4.8676
2	−4.8397	−4.3634	29.595	0	0	−4.3182
3	0	0	−58.96	—	—	—

### Parameter selection of the equilibrium geometry model

The parameters of equilibrium geometry model include melt composition, basalt fraction, reference potential temperature and dihedral angle of melt. The melt composition and basalt fraction are the two most important factors affecting the solid elasticity.

#### The bulk moduli and high pressure densities of the different melt compositions

The partial melting in the deep upper mantle is carried out with the participation of H_2_O which is easier into melt than the residual solid [24], so the melt is hydrous. The partial melting of the peridotite in dry and wet conditions at the bottom of the upper mantle results in the formation of ultrabasic melts [[25], [26], [27]], which differ with the basic melts in shallower mantle [28]. As the same, the carbonated peridotite can produce the partial melt at the bottom of the upper mantle [29,30]. So the hydrous peridotite or carbonated peridotite is more representative partial melting product at the bottom of the upper mantle. The equation of state of melts are applied to calculate the densities under high pressure conditions. The 3rd Birch-Murnaghan equation of state is adopted in this paper:

(20) $$P=\frac{3}{2}K_{T_{0}}\{{\left(\frac{\rho }{\rho_{0}}\right)}^{\frac{7}{3}}-{\left(\frac{\rho }{\rho_{0}}\right)}^{\frac{5}{3}}\}[1-\frac{3}{4}(4-K_{T_{0}}^{'})\{{\left(\frac{\rho }{\rho_{0}}\right)}^{\frac{2}{3}}-1\}]$$

where *K*_T0_ and $K_{{\text{T}}_{0}}^{\prime }$ are the isothermal bulk modulus and its pressure derivative. *ρ*_0_ and *ρ* are the densities at room and high pressures.

The bulk modulus can be expressed as:

(21) $$K=K_{T_{0}}+K_{T_{0}}^{'}\cdot P$$

Table 3 lists the bulk moduli and density of melts under the pressures at the Earth’s surface and atop the LVL.

**Table 3:** The bulk moduli and density of melts under the pressures at Earth’s surface (a) and atop the LVL (b).

(a)				
Melt composition	K_0_(GPa)	*ρ*_0_(10^3^kg/m^3^)	K′	References
MORB	15.5	2.59	7.2	Guillot and Sator, 2007
Peridotite (IT8720)	32.0	2.87	4.6	Sakamaki et al., 2006
IT8720 + 2 wt.%H_2_O	19.5	2.69	5.8	Sakamaki et al., 2006
IT8720 + 8 wt.%H_2_O	6.9	2.24	7.2	Sakamaki et al., 2006
Carbonated peridotite	24.9	2.67	5.1	Ghosh et al., 2007

(b)			
Melt composition	P(GPa)	*ρ*(10^3^kg/m^3^)	K(GPa)
MORB	11.7	3.4419	99.74
IT8720		3.5754	85.82
IT8720 + 2 wt.%H_2_O		3.5352	87.36
IT8720 + 8 wt.%H_2_O		3.3960	91.14
Carbonated peridotite		3.4234	84.57

#### Basalt fraction

The partial melting at mid-ocean ridges generates a basaltic crust and leaves behind the depleted complement, harzburgite, thus the oceanic lithosphere is physically and chemically layered and continuously injects into the mantle during slab subducting. The mantle should be considered as a non-equilibrated mechanical mixture of basalt and harzburgite [31]. Following the self-consistent thermodynamic model developed by Stixrude and Lithgow-Bertelloni [32,33], Xu et al. [31] computed the seismic velocities of the Equilibrium Assemblage (hereafter as EA) with perfect equilibration and the Mechanical Mixture (hereafter as MM) with perfect disequilibrium between the two fractions. They calculated the density, compressional wave velocity (V_P_), and shear wave velocity (V_S_) of EA and MM with basalt fraction varying from 0 % to 100 % and along adiabats with potential temperatures ranging from 1000 K to 2000 K. The database (See Table 3 of Appendix from Xu et al. [31]) covers a wide range of areas including the whole mantle.

Based on the EA data from Xu et al. [31], the same range 0∼40 vol. % of basalt fraction with Hier-Majumde et al. [34] were used in this paper.

#### Reference potential temperatures

Convection is considered to be the main mode of heat conduction in the mantle under the lithosphere, and the temperature gradient is very close to the adiabatic gradient caused by the mantle convection. The thermodynamic relationship between entropy of unit mass, temperature and pressure can be expressed as:

(22) $$dS=\frac{c_{P}}{T}dT-\frac{\alpha_{V}}{\rho }dP$$

where *c_P_* is the heat capacity at constant pressure, and *α_V_* is the volume thermal expansion coefficient. In the reversible adiabatic process, the change of entropy is zero: *d_S_* = 0. So the Eq. (22) can be translated into:

(23) $${\left(\frac{dT}{dP}\right)}_{S}=\frac{\alpha_{V}T}{\rho c_{P}}$$

The increase in pressure with depth may be expressed as:

(24) $$\frac{dP}{dy}=\rho g$$

Where *g* is gravity acceleration.

And the adiabatic temperature gradient can be obtained by multiplying the two Eqs. (23) and (24):

(25) $${\left(\frac{dT}{dy}\right)}_{S}=\frac{\alpha_{V}gT}{c_{P}}$$

If$\frac{\alpha_{V}g}{c_{P}}$, the equation is integrated as:

(26) $$T=T_{0}e^{Ay}$$

where *T*_0_ is an adiabatic expansion to a surface where the pressure is zero, which also is called the potential temperature.

**Fig. 2:**
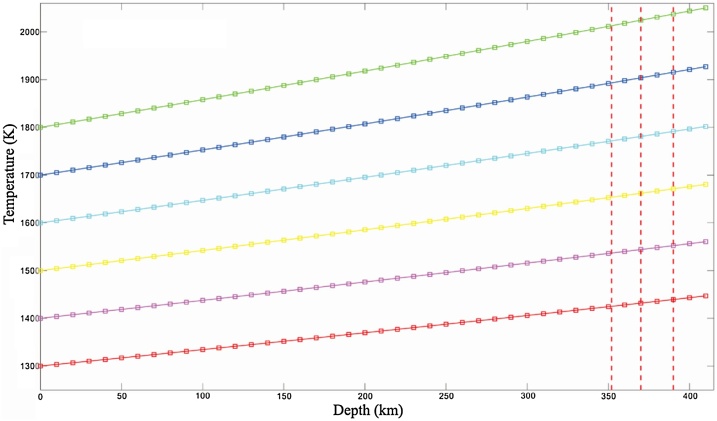
Adiabatic temperature line for basalt fraction 0 (1300−1800 K). Lines in different colors represent different adiabatic temperatures, where blue ones are for 1800 K, dark blue for 1700 K, light blue for 1600 K, yellow for 1500 K, purple for 1400 K, red for 1300 K.

**Fig. 3:**
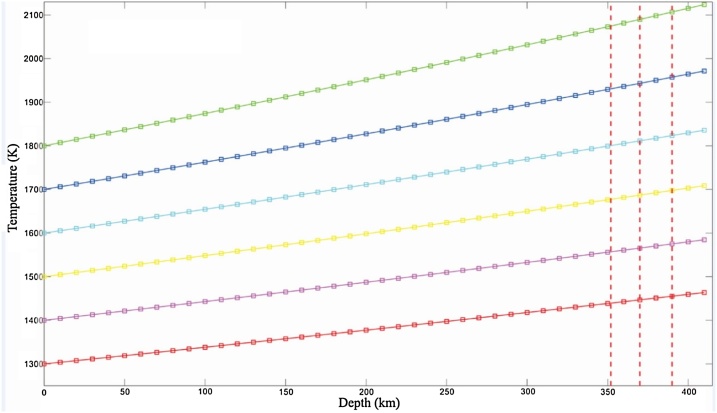
Adiabatic temperature lines for basalt fraction 0.20 (1300−1800 K). More descriptions are same as Fig. 2.

Since *c_P_* and *α_V_* are related with composition parameters, *A* is also associated with composition. Using the database of Xu et al. [31], one depth can be chosen for an adiabatic temperature of the same composition, corresponding entropy value at the depth is the same as the entropy value at the surface (e.g., 1300 K). Then taking the depth and temperature data into the Eq. (26), the value of *A* and the mantle adiabatic temperature lines of this composition can be obtained (Fig. 2, Fig. 3). Lastly, the corresponding adiabatic temperatures can be obtained by substituting the LVL depths to equation of the adiabatic temperature line of the mantle.

#### Dihedral angle of melt

The dihedral angle is an important parameter to describe the property of melt, which can significantly affect melt volume fraction [35].

Under low pressure conditions (≤3 GPa), the dihedral angles range between 20° and 50° for basaltic melts [36] and between 25° and 30° for carbonated melts [37], 28 ± 3° for hydrous basalt melt [38], and 25°∼30° for carbonate melt and olivine [37]. From lower pressure experiments, the dihedral angles monotonically decrease with pressure increasing, even become 0° above at least 7 GPa [39]. Since the pressure atop the MTZ is 13−14 GPa [40], the dihedral angles of melts may be lower, e.g., 0°–10°. Therefore, 5° can be taken as the lower limit of dihedral angles and 30° corresponding to the hydrous basalt melt and carbonate melt can be taken as the upper limit because the partial melting atop the MTZ takes place with the participation of volatiles, such as H_2_O and CO_2_.

### Reference solid elastic properties atop the LVL

The database of Xu et al. [31] tabulates the physical properties of solid directly, such as $V_{\text{S}}^{0}$ 、 $V_{\text{P}}^{0}$ and ρ on increments of 100 K. For a certain composition and a certain temperature, $V_{\text{S}}^{0}$ 、 $V_{\text{P}}^{0}$ and ρ corresponding to the top temperature of the LVL is interpolated according to the third order polynomial used by Hier-Majumder et al. [34] and the remaining parameters such as *μ*, *k* and *υ* can be expressed in the following three Eqs. (27), (28), (29):

(27) $$\mu ={\left(V_{\text{S}}^{0}\right)}^{2}\cdot \rho$$

(28) $$K=\left[{\left(V_{\text{P}}^{0}\right)}^{2}-\frac{4}{3}{\left(V_{\text{S}}^{0}\right)}^{2}\right]\cdot \rho$$

(29) $$\nu =\frac{{\left(\frac{V_{\text{P}}^{0}}{V_{\text{S}}^{0}}\right)}^{2}-2}{2\left[{\left(\frac{V_{\text{P}}^{0}}{V_{\text{S}}^{0}}\right)}^{2}-1\right]}$$

Based on the above analysis, it can be concluded that the melt fraction equation of poroelastic effect belongs to the category of nonlinear algebraic equation. The problem can be solved with the least square method.
